# Endorsement of the Proposition for the State to Control Procreation of the Defective Classes

**Published:** 1913-03

**Authors:** 


### Endorsement of the Proposition for the State to Control Procreation of the Defective Classes.

The American Institute of Criminal Law and Criminology, recently held in Milwaukee, considered the propositions:

1. In what ways can propagation of habitual criminals, imbeciles and lunatics be prevented?

2. Should sterilization of such persons in proper cases be authorized by law?

The committee reporting on the above questions stated in part:

There are three methods by which the propagation of thees defective classes can be influenced: by regulation of marriage, by effective sequestration, and by destroying the power of the individual to produce offspring.

Laws have been passed attempting to regulate marriage. There is no doubt but that conditions have been somewhat improved by such laws. It is wise to withdraw the approval of the State from the marital union of persons unfitted to [bear] care for and train the children they might produce.

Sequestration, if perfect, would be absolutely effective. It is doubtful if it can be made complete, however, especially in men. Among the insane, parole, in some cases, appears not only justifiable but beneficial[?]. Among the feeble-minded, absolute enforcement of sequestration would appear unwise. In such cases it would unnecessarily tend to separate some children from their families permanently; and, moreover, the higher grades of the feeble-minded are instinctive tramps, and absolute sequestration would mean a closeness of confinement totally at variance with the idea that institutions should be colonies, with almost the freedom of action of the country village, rather than places of confinement.

The jury trial is always available, and it is difficult to make the average jury believe that there is just cause for retention under the care of the State, of high-grade imbeciles, who are often of prepossessing appearance, especially when the moral qualities are chiefly at fault. With girls, retention is easier than with boys, as their tendencies for elopement are not so marked.

It cannot be doubted that sequestration is, in some measure, effective. For the last few years a steady decrease has been noted in the number of applications to the Wisconsin Home for Feeble-Minded, due, the committee believes, to the great number of highest grade feeble-minded women who have been retained under the shelter of the institution.

In most of the few cases in which the higher grade girls have been taken from State care, the results have been regrettable, but conditions have been better than in some States in which the control of the State is nominal. The superintendent of one institution asserts that he is now receiving the children of the girls released.

The number of feeble-minded, including juvenile delinquents (the criminal feeble-minded), is enormous. Dr. Goddard of the department of research in the New Jersey institution estimates the present number in the United States at 300,000. He points out, especially, the large proportion of feeble-minded in reform schools, placing them at not less than 25 per cent. These are the "criminals coming within the scope of the committee's activity," because their condition is permanent. They are usually sufficiently bright to mate with others of about the same mental status, and begin new lines of degeneracy. Unless they are under guardianship, they do not have the concentration of purpose to make a living for themselves and those dependent on them, or judgment to prefer rightdoing to wrongdoing, or the will-power to resist the ordinary temptations of life, which come to all. Punishment has little effect on them, and reasoning is wasted. They are "repeaters" in crime. They are generally the offspring of those who have weakened their moral control through constant self-indulgence. They would probably belong in the class considered by the committee as proper subjects for surgical interference. From them spring all forms of neurotic defects.

For the purpose now under consideration, feeble-mindedness is the most important subject, and with it epilepsy may properly be classed, as feeble-minded or epileptic parents almost invariably transmit their qualities to their children in varying degrees; such offspring, on account of their incapacities, misdeeds or crimes, frequently become public charges or come into conflict with the authorities of the law.

Certain types of insanity, notably dementia precox and manic-depressive insanity in the parents, show a marked tendency to reproduce themselves in the children as feeble-mindedness, criminality, and other forms of degeneracy.

Neurotics reproduce neurotics with tendencies to neuroses, insanity, feeble-mindedness, drunkenness, pauperism and criminality. Insanity is more often inherited from the insane. Feeble-mindedness is the result of all neuroses, since any check to the growth of the brain must affect the development of the mind. In short, except in rare instances, feeble-mindedness, insanity, criminality, inebriety and pauperism may be looked on as allied conditions with marked tendencies to be reproduced in one or another form in the children of persons affected with such infirmities, and with a strong tendency for the defects to become more marked with each succeeding generation and to be accentuated by unions between such defectives, as persons belonging to these various classes have a strong affinity for others of their kind.

Efforts have been made to find a family in which two defectives have united and produced normal children. No record of such a family has been found.

That the insane are increasing much faster than the general population is undoubted. The census reports show this. The increase has aroused public interest to such an extent that several States have passed laws for the absolute prevention of increase by operation. The decision in each State is left to a commission.

